# Supplementary material for: Beyond emotions: Social cognitive predictors of COVID-19 vaccination intentions before and after vaccine roll-out
Source: PLOS Glob Public Health. 2026 Jan 2;6(1):e0005668. doi: 10.1371/journal.pgph.0005668 (PMC12758815; doi:10.1371/journal.pgph.0005668)
Supplement: Attachment — Submitted filename: Response to Reviewers.docx [file pgph.0005668.s002.docx]

We thank the editor and all reviewers for their thoughtful, and constructive feedback. Their comments have greatly helped us improve the clarity, coherence, and impact of the manuscript. We have carefully considered each point and made corresponding revisions throughout the paper. Our detailed responses are provided in red below, directly addressing each reviewer’s comment point by point.

**Comments to the Author**

5. Review Comments to the Author

Reviewer #1: No comments. The paper is well written and all the different areas including introduction, materials and methods, results, data analysis and discussions are well written and well articulated. The paper covers all areas well and succintcly.

We sincerely thank Reviewer #1 for the positive evaluation and encouraging feedback. We are very pleased that the manuscript was found to be clearly written, comprehensive, and well-structured across all sections.

Reviewer #2: 1. The intro section could be tightened slightly by reducing redundancy in references describing early-pandemic hesitancy trends and focusing on what remains unknown or understudied..specifically, within-person change over time, recommend adding these new studies which cover this perspective

We thank the reviewer for this suggestion. The Introduction was revised to remove repetitive descriptions of early-pandemic vaccination-hesitancy findings and overlapping reference clusters. Redundant examples of risk, attitude, and safety predictors were condensed. The revised text now places greater emphasis on the under-studied within-person perspective by explicitly highlighting the lack of longitudinal, multi-country research examining how social-cognitive and emotional determinants of vaccination intentions evolve across pandemic phases. We also integrated recent longitudinal evidence to illustrate this gap. The new paragraph beginning “Despite this growing evidence, most research has relied on cross-sectional data…” (page 6, paragraph 6) introduces this focus and frames the study’s unique contribution as a direct within-person comparison of predictors before and after vaccine rollout.

2. Strengthen the discussion to address possible cross-cultural differences in psychosocial predictors, given the study’s multinational scope
Additionally, recommend citing <https://doi.org/10.1136/bmjph-2025-002765>, new 2025 study which provides complementary evidence on population-level disruption and recovery during the pandemic, aligning well with the observed declines in self-efficacy and pro sociality reported in this manuscript

We thank the reviewer for this constructive suggestion. A new paragraph was added to the Discussion (immediately before the “Strengths and Limitations” section) to address potential cross-cultural variation in psychosocial predictors. This addition discusses how cultural orientation, institutional trust, and policy context may influence self-efficacy, prosociality, and vaccination intentions across countries. We also incorporated the 2025 BMJ Public Health article (Balsara et al., 2025) as complementary population-level evidence of psychosocial disruption and uneven recovery during the pandemic. This reference is used to contextualise our findings of reduced self-efficacy and prosociality within broader patterns of societal adaptation and recovery.

3. The results section could be improved by adding a short statement on effect sizes or confidence intervals for the main predictors, enhancing interpretability and policy relevance

We thank the reviewer for this helpful observation. The Results section already reported odds ratios and 95% confidence intervals for all key predictors; however, these have now been highlighted more clearly in the text to enhance interpretability and policy relevance. Specifically, brief quantitative statements were added to summarise the magnitude of the main effects (e.g., self-efficacy was associated with 30–36% greater odds of vaccination intention). These clarifications improve the readability of the Results section and make the strength and precision of the key predictors more immediately interpretable for readers.

4. Consider clarifying whether psychological flexibility and pro sociality were treated as independent or interrelated constructs in the models, as their conceptual overlap could influence mediation pathways

Psychological flexibility and prosociality were treated as independent predictors within all cumulative link models. Although these constructs are conceptually related, correlation analyses indicated no problematic overlap (r < .60; see Table 1), and both variables were retained as distinct predictors. A clarifying sentence has been added to the Statistical Analysis section to make this analytic approach explicit.

5. A brief mention of pandemic fatigue or behavioral adaptation literature (e.g., sustained stress leading to reduced self-efficacy) could provide a more nuanced interpretation of the post-rollout decline in psychosocial measures

The Discussion section has been revised to incorporate relevant literature on pandemic fatigue and behavioural adaptation to contextualise the observed post-rollout decline in psychosocial measures. Specifically, new references have been added highlighting how sustained stress, uncertainty, and prolonged adherence demands can diminish motivation, perceived behavioural control, and collective engagement over time.

6. Regarding the data availability statement, it currently notes that access will be provided to “researchers who meet the eligibility criteria.” This phrasing is vague and does not fully align with the journal’s open-data policy. The authors should clarify the specific access conditions, including who determines eligibility and how qualified researchers can request the dataset

We appreciate the reviewer’s feedback and have revised the Data Availability Statement for greater clarity and transparency. The updated statement now specifies that the fully anonymised dataset is available through the Open Science Framework (OSF) at the provided link, ensuring compliance with the journal’s open-data policy.

7. Tables 2–4 would benefit from clearer labeling of model variables and consistent reporting of p-values and ORs to improve readability

Tables 2–4 have been revised to improve clarity and consistency. Variable labels have been standardised to match terminology used in the text, and table captions have been simplified for readability. All p-values are now italicised and reported using consistent decimal precision, and odds ratios (ORs) and 95% confidence intervals are formatted uniformly across models. Explanatory notes have been added below each table to clarify variable definitions, reference categories, and statistical tests. These adjustments enhance the overall readability and ensure that tables align with journal reporting standards.

Reviewer #3: The manuscript, titled 'Beyond Emotions: Social Cognitive Predictors of COVID-19 Vaccination Intentions Before and After Vaccine Roll-Out,' appears to meet the publication criteria by describing methodologically rigorous research. The study utilised a multinational prospective design across 33 countries, examining social-cognitive and emotional predictors of COVID-19 vaccination intentions before and after vaccine rollout. This approach allows for comparisons across two distinct phases of the pandemic, enhancing the robustness of the findings. The conclusions drawn, particularly regarding the consistent predictive power of self-efficacy and perceived severity, are directly supported by the data presented. The study also highlights declines in self-efficacy, pro-sociality, psychological flexibility, and positive affect after vaccination rollout, which aligns with evidence of pandemic-related psychological fatigue.

The study was approved by the Cyprus National Bioethics Committee (ΕΠ 2020.01.60), and all participants provided informed consent electronically before participation, indicating ethical conduct.

The statistical analysis appears to be performed appropriately and rigorously. Changes in psychological variables between the pre-rollout (Time 1) and post-rollout (Time 2) phases were assessed using paired t-tests. To predict vaccination intention, cumulative link models (CLMs) were fitted, treating vaccination intention as an ordinal outcome, and estimated using the 'ordinal' package in R. Four models were specified, progressively incorporating psychological predictors, sociodemographic covariates, and COVID-19 infection status variables. Intercorrelations among predictors were examined to assess multicollinearity, with no problematic associations found (r < .60). This detailed approach suggests a comprehensive and rigorous statistical methodology.

The manuscript explicitly states that the data contain potentially identifying or sensitive participant information and cannot be shared publicly. Instead, data are available from the corresponding author for researchers who meet the criteria for access to confidential data. This aligns with PLOS policy for sensitive data, where full public availability might be restricted due to ethical concerns. The authors have provided a transparent statement regarding data access.
The manuscript is presented in an intelligible fashion and generally written in standard English. The language is clear, and the structure is logical, making the content easy to follow. No significant typographical or grammatical errors were noted that would impede understanding.

The study's multinational, prospective design is a significant strength, allowing for a robust examination of vaccination intentions across different phases of the pandemic and diverse populations. The identification of self-efficacy and perceived severity as consistent predictors of vaccination intention, even as other psychosocial factors declined, provides valuable insights for public health messaging.

We sincerely thank Reviewer #3 for the highly positive and encouraging assessment of our manuscript. We appreciate the recognition of the study’s methodological rigour, clear presentation, and contribution to understanding psychosocial and social-cognitive predictors of vaccination intentions. We are particularly grateful for the reviewer’s acknowledgement of the robustness of our statistical approach, the clarity of the writing, and the value of our findings for public health communication.

Suggestions for Improvement:

Generalisability of Sample: While the study includes a diverse, cross-national sample, the authors acknowledge that the sample was predominantly female, which may limit generalizability. Further discussion of how this demographic imbalance might influence the findings, or how future research could address it, would be beneficial.

The Discussion section has now been expanded to address how the predominantly female composition of our sample may influence generalisability. Recent evidence suggests that women have shown slightly higher vaccine hesitancy and lower uptake in several contexts (Robertson et al., 2022; Yam et al., 2024), which may have led to somewhat lower overall vaccination intentions in our sample. We have now acknowledged this potential bias and noted that future studies should aim for gender-balanced recruitment and examine potential gender differences in psychosocial predictors of vaccination behaviour.

Perceived Susceptibility: The finding that perceived susceptibility was negatively associated with vaccination intentions before rollout, but not after, is intriguing. The explanation provided (anxiety, mistrust, or concerns about vaccine safety when vaccines were not yet available) is plausible. Expanding on this point, perhaps with a brief discussion of how public health communication could specifically address these early concerns about susceptibility and safety, could add practical value.

The Discussion section has been refined to elaborate on the negative association between perceived susceptibility and vaccination intentions before rollout. The revised text explains that, although perceived susceptibility typically promotes preventive behaviour by heightening personal relevance and risk salience, during the early pandemic phase it may have amplified anxiety and uncertainty when vaccine information was limited and mixed messages circulated. We further note that transparent, trust-building communication about vaccine safety and efficacy can help restore the positive motivational impact of risk perception.

Psychological Variables as Non-Significant Predictors: The manuscript states that other psychological variables (e.g., perceived stress, psychological flexibility, pro-sociality, positive and negative affect) were not significant predictors of intentions. Given that some of these factors declined post-rollout, a more in-depth discussion on why they did not predict intention, despite their shifts, could provide further nuance to the findings.

The Discussion section has now been expanded to further explain why several psychological variables did not significantly predict vaccination intentions despite declining after the rollout. The revised text clarifies that although factors such as perceived stress, psychological flexibility, and prosociality reflect broader emotional adaptation to the pandemic, their limited predictive power may indicate a shift from general affective responses to more specific social-cognitive drivers of behaviour once vaccines became available. We also note that these general psychological variables may have been statistically overshadowed by more proximal, vaccine-specific cognitions—such as perceived safety, confidence, collective responsibility, and trust in authorities—which tend to capture greater variance in vaccination intentions. This interpretation is supported by recent evidence showing that belief-based and informational factors outweigh general psychological traits in explaining vaccine hesitancy (Charron et al., 2021; Annandale et al., 2025). Together, these additions provide a more nuanced theoretical and empirical interpretation of both significant and non-significant predictors.

Finally, the manuscript presents methodologically sound and ethically rigorous research, with conclusions well-supported by the data. The statistical analysis is appropriate, and the data availability statement is clear. The paper is well-written, and the suggested improvements aim to further enhance the depth and practical implications of the findings.

We thank the reviewer for the positive and encouraging assessment of the manuscript’s methodological rigour, clarity, and ethical standards. We appreciate the acknowledgement that the analyses and conclusions are well supported by the data and have carefully implemented the suggested improvements to further strengthen the paper’s depth and practical relevance.
